# Electroactive and Thermoresponsive Hybrid Microgel on a Gold Surface for Electrochemically Controlled Release of Active Substance

**DOI:** 10.1021/acsami.5c06165

**Published:** 2025-06-13

**Authors:** Paulina Gwardys, Kamil Marcisz, Damian Jagleniec, Jan Romanski, Marcin Karbarz

**Affiliations:** † 49605 University of Warsaw, Faculty of Chemistry, 1 Ludwika Pasteura Str., PL 02-093 Warsaw, Poland; ‡ 49605 University of Warsaw, Biological and Chemical Research Center, 101 Żwirki i Wigury Av., PL 02-089, Warsaw, Poland

**Keywords:** “smart microgel”, PEDOT, microgel monolayer, electrochemically controlled release, crystal violet

## Abstract

In this study, we present a thermoresponsive and electroactive hybrid microgel immobilized on an electrode surface, designed as a platform for electrochemically induced release of positively charged model substances. The microgel was synthesized using *N*-isopropylacrylamide (NIPA) copolymerized with sodium acrylate (AcNa) and cross-linked with a cystine derivative (BISS). Poly­(3,4-ethylenedioxythiophene) (PEDOT) spheres were incorporated within the microgel matrix, enhancing its electroactive properties. The structural and functional properties of the microgel were characterized using dynamic light scattering (DLS), transmission electron microscopy (TEM) coupled with energy dispersive spectroscopy (EDS), and cyclic voltammetry. The microgel was anchored onto the gold electrode surface through a chemisorption process facilitated by disulfide bridges present in the cross-linking agent. The adsorption process was monitored using quartz crystal microbalance with energy dissipation (QCM-D). Subsequently, a positively charged model compound, crystal violet, was incorporated into the microgel structure through electrostatic interactions with carboxyl groups. The electrochemically induced release of the dye molecules was then investigated. Upon applying an electrochemical potential to the microgel-coated electrode, oxidation of PEDOT groups generated positive charges. This, in turn, disrupted the electrostatic interactions between the microgel network and the dye molecules, facilitating their release into the surrounding medium. The release process was quantitatively monitored using UV–Vis spectrophotometry. This system demonstrates potential as a promising drug delivery platform for use in implantable biomedical devices.

## Graphical abstract

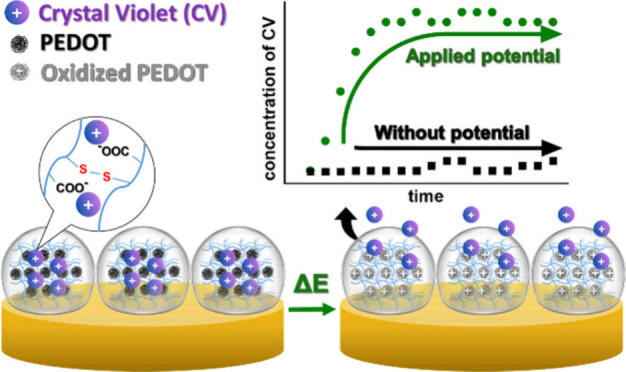

## Introduction

Polymer microgels are spherical structures with dimensions comparable to colloidal particles. They consist of a three-dimensional polymer network that swells in the solvent in which they are dispersed.^1–3^ These materials are notable for their high surface area and low viscosity, making them highly versatile.^4^ Microgels are classified as “smart” materials due to their responsiveness to various environmental factors. One particularly intriguing property of microgels is their ability to undergo a volume phase transition (VPT), a reversible change in volume triggered by external stimuli such as temperature, pH, ionic strength, light, or electric fields.^5–9^

A notable subgroup of microgels comprises electroactive systems, which either incorporate electroactive compounds within the polymer network or are entirely based on conductive polymers. These materials exhibit VPT behavior in response to changes in the oxidation state of the gel network. Electroactive compounds can be integrated into microgels via covalent bonding or electrostatic interactions. In the covalent method, redox-active molecules are chemically attached to the polymer network, and their oxidation state alters the hydrophilic/hydrophobic balance, thereby affecting the microgel’s volume.^10,11^ In contrast, the electrostatic approach leverages interactions between electroactive groups and polymer chains, where changes in oxidation state modify the strength of multipoint interactions.^12,13^ Electroactive microgels tethered to conductive surfaces have been investigated for applications such as sensors, biosensors, logic gates, and advanced drug delivery systems.^14–19^

Microgels exhibit significantly faster and more pronounced swelling behavior compared to regular-sized hydrogels, which makes them particularly promising for controlled drug delivery applications.^4,20^ Among these, poly­(*N*-isopropylacrylamide) (pNIPA)-based microgels are the most widely studied due to their thermosensitivity. These microgels possess a volume phase transition temperature (VPTT) of approximately 32 °C. Above the VPTT, pNIPA microgels transition from a swollen to a shrunken state. Modifications to the polymer network can further tune the VPTT, enhancing their functionality.^4,20–24^ Another extensively researched class of microgels includes pH-sensitive systems, such as those based on poly­(acrylic acid) (pAA). These microgels adjust their swelling behavior in response to environmental pH changes, enabling the targeted release of encapsulated agents in specific body regions with distinct pH levels. This property enhances therapeutic efficacy while minimizing side effects.^25–27^

Multiresponsive microgels, as “smart” materials that respond to multiple stimuli, have emerged as powerful tools for biomedical applications, particularly in drug delivery.^28,29^ They enable controlled drug release through finely tuned physicochemical interactions, which can be modulated using external stimuli such as voltage, light, or temperature.^30–34^ When assembled on conductive surfaces, these systems offer precise spatial and temporal control over the release of both small and large molecule drugs. For example, in a study by Xu et al., a microgel layer composed of poly­(*N*-isopropylacrylamide-*co*-acrylic acid) was fabricated on an electrode surface and employed as an electro-responsive release platform. Positively charged dye molecules were incorporated into the negatively charged polymer matrix via electrostatic interactions. Upon application of a suitable reduction potential, electrolysis of water occurred, leading to a localized decrease in pH near the electrode. This pH shift resulted in protonation of the carboxylic acid groups, weakening the electrostatic interactions and triggering the release of the dye molecules from the microgel network.^35^ Similarly, Wang et al. demonstrated a wireless implant system for the electrochemically induced release of fluorescein, utilizing a polypyrrole nanoparticle-based conductive film. In this case, negatively charged fluorescein was loaded into a positively charged polymer matrix and released upon reduction of the conductive layer.^36^ Furthermore, hydrogels exhibit tunable physical properties and can protect labile drugs from degradation while maintaining responsiveness to external stimuli.^37–39^

Among conducting polymers, poly­(3,4-ethylenedioxythiophene) (PEDOT) stands out due to its exceptional properties. PEDOT is highly stable under harsh doping conditions, exhibits superior electrical conductivity compared to most polymers, and is biocompatible, facilitating seamless interfacing with biological systems such as cells and tissues. Additionally, PEDOT is relatively easy to synthesize, making it a highly promising platform for the controlled release of therapeutic agents.^40–43^ The tunable drug delivery capabilities of PEDOT are driven by electrically induced changes in its redox state, which alter key properties such as volume, charge, and hydrophilic/hydrophobic balance. Coupling PEDOT moieties with environmentally sensitive microgels resulted in the formation of a multiresponsive material with unique properties.^44,45^ Despite their potential, PEDOT-based hydrogels have been underexplored in drug delivery applications.^43,46^ Nonetheless, relatively few studies have investigated their application in this area, even though they exhibit several advantageous properties. For instance, Chika et al., who developed a double-coating system combining an RGD-functionalized alginate hydrogel with a PEDOT-coated electrode, enabling trophic factor release upon electrical stimulation.^47^ Similarly, Molina et al., who studied PEDOT nanoparticles encapsulated in a poly-γ-glutamic acid biohydrogel for vitamin K3 release. Using differential pulse voltammetry, they demonstrated precise drug loading and release, paving the way for theragnostic therapies.^48^ Additionally, Puiggalí-Jou et al., who explored the electro-induced release of curcumin, a hydrophobic drug, from PEDOT/alginate-based hydrogels, showcasing the versatility of these systems.^49^

In this study, a thermosensitive hybrid microgel based on NIPA containing PEDOT was synthesized and evaluated as an electrochemically controlled drug delivery platform. The hydrogel was synthesized via precipitation polymerization, employing an acrylate derivative of cystine as a cross-linker. This cross-linker introduced disulfide bridges into the structure, enabling the microgel to anchor onto gold electrode surfaces via chemisorption. Sodium acrylate was used as a copolymer to introduce negative charges into the polymer network, while PEDOT nanoparticles were immobilized within the microgel network as electroactive centers. The negatively charged groups facilitated the immobilization of a model substance, crystal violet (CV), a positively charged dye molecule. The electrochemically induced release of CV from the microgel-modified electrode surface was investigated, demonstrating the system’s potential for precise, stimuli-responsive drug delivery.

## Materials and Methods

### Materials

*N*-Isopropylacrylamide (NIPA), sodium acrylate (AcNa), 3,4-ethylenedioxythiophene (EDOT), potassium persulfate (KPS) and crystal violet (CV) were purchased from Aldrich. Sodium hydroxide (NaOH), hydrochloric acid (HCl), sodium nitrate (NaNO_3_), sulfuric acid (H_2_SO_4_), hydrogen peroxide (H_2_O_2_) and acetonitrile (ACN) were purchased from POCh. *N*,*N*′-bisacryloylcystine (BISS) was synthesized according to the literature procedure.^20^ All reagents were used as supplied, except for NIPA, which was purified by recrystallization from a 3:7 (v/v) toluene/hexane mixture. All solutions were prepared using high purity water obtained from a Milli-Q Plus/Millipore purification system (water conductivity: 0.056 μS cm^–1^).

To synthesize *N,N’*-bisacryloylcystine, the following procedure was utilized: a solution containing sodium hydroxide and cystine in methanol underwent stirring, and acryloyl chloride was cautiously added dropwise at 0 °C. The resultant solution was further stirred at ambient temperature. After a duration of approximately 4 h, the reaction mixture was subjected to filtration using a Celite pad. The filtrate was then gradually introduced dropwise into vigorously stirred cold diethyl ether. The resulting suspended solid was separated through filtration, treated with diethyl ether washing, and subsequently dried using high vacuum conditions within the range of 30–45 °C. The analysis based on sulfur content from combustion analysis revealed the presence of approximately 65% of the disodium salt of *N*,*N*′-bisacryloylcystine in the powder. The successful synthesis of *N,N’*-bisacryloyl cysteine was confirmed using ^1^H NMR, ^13^C NMR and mass spectroscopy techniques.

### Synthesis of Poly­(3,4-ethylenedioxythiophene) (PEDOT) Conductive Polymer/Nanoparticles

PEDOT was synthesized via the oxidative polymerization of EDOT monomers (Figure 1), following a method described in the literature.^45^ Briefly, 74 μL of EDOT was dissolved in 2 mL of ethanol and subsequently mixed with 4 mL of Milli-Q water. Then, 140 mg of the oxidizing agent, FeCl_3_·6H_2_O dissolved in 4 mL of water, was added to the EDOT solution. Potassium persulfate (KPS) may also act as an initiator in the EDOT polymerization reaction. Compared to commonly used iron­(III), KPS offers a different oxidation pathway, which may influence the resulting polymer’s properties, including conductivity and morphology.^50^ The mixture was stirred at room temperature for 36 h using a magnetic stirrer, during which a color change from colorless to dark blue was observed, indicating the formation of PEDOT. In the next step, the obtained PEDOT particles were centrifuged at 6000 rpm for 10 min and then washed with methanol to remove unreacted compounds. Subsequently, the centrifuged PEDOT was dispersed in water using ultrasonic treatment for 10 min. The resulting dispersion was transferred to a dialysis membrane with a pore size of 10 kDa and placed in a 5 L container filled with Milli-Q water for several days to remove residual iron ions, with the water being replaced daily.

**Figure 1:**
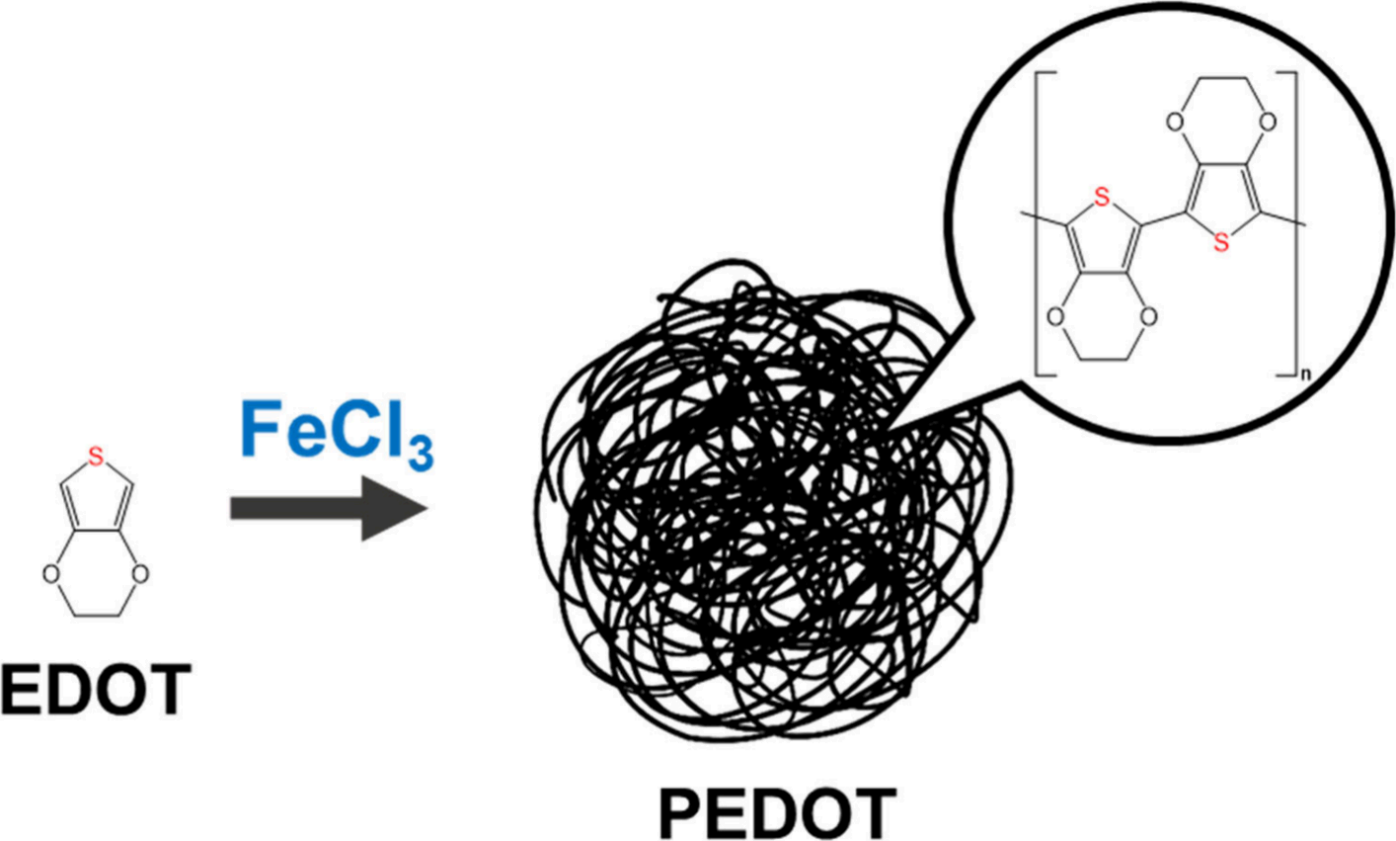
Scheme of PEDOT synthesis.

### Synthesis of PEDOT-Functionalized p­(NIPA-BISS-AA) Microgel

The microgel was synthesized using a precipitation polymerization method, as illustrated in Figure 2. A 10 mL monomer solution with a final concentration of 70 mM, consisting of 90% NIPA, 3% BISS, and 7% AcNa, was prepared in water. Subsequently, 5 mL of PEDOT dispersed in water was added to the mixture. The solution was purged with argon gas for approximately 40 min to remove oxygen. After deoxygenation, the solution was heated to 70 °C and stirred at 200 rpm using a magnetic stirrer. At this stage, 5 mL of a KPS initiator solution was added to initiate the polymerization reaction. The polymerization process was carried out under an argon atmosphere for 3 h, after which the solution was allowed to cool to room temperature. The obtained microgel was then transferred to a dialysis membrane with a 10 kDa pore size and dialyzed in a 5 L container filled with Milli-Q water to remove unreacted components. The dialysis process was carried out over 6 days, with the water replaced daily. After synthesis, the obtained microgel was analyzed using nuclear magnetic resonance (NMR) spectroscopy. To prepare the sample, the microgel was lyophilized, dissolved in D_2_O, and then examined. A characteristic signal for PEDOT moieties was observed at 4.97 ppm, while characteristic signals for polymeric network based on p­(NIPA-AA) were detected at 0.91–1.24, 1.31–1.79, 1.81–2.22, and 3.82 ppm.^51,52^ Characteristic signals from BISS were not well visible due to the small content of the cross-linker.

**Figure 2:**
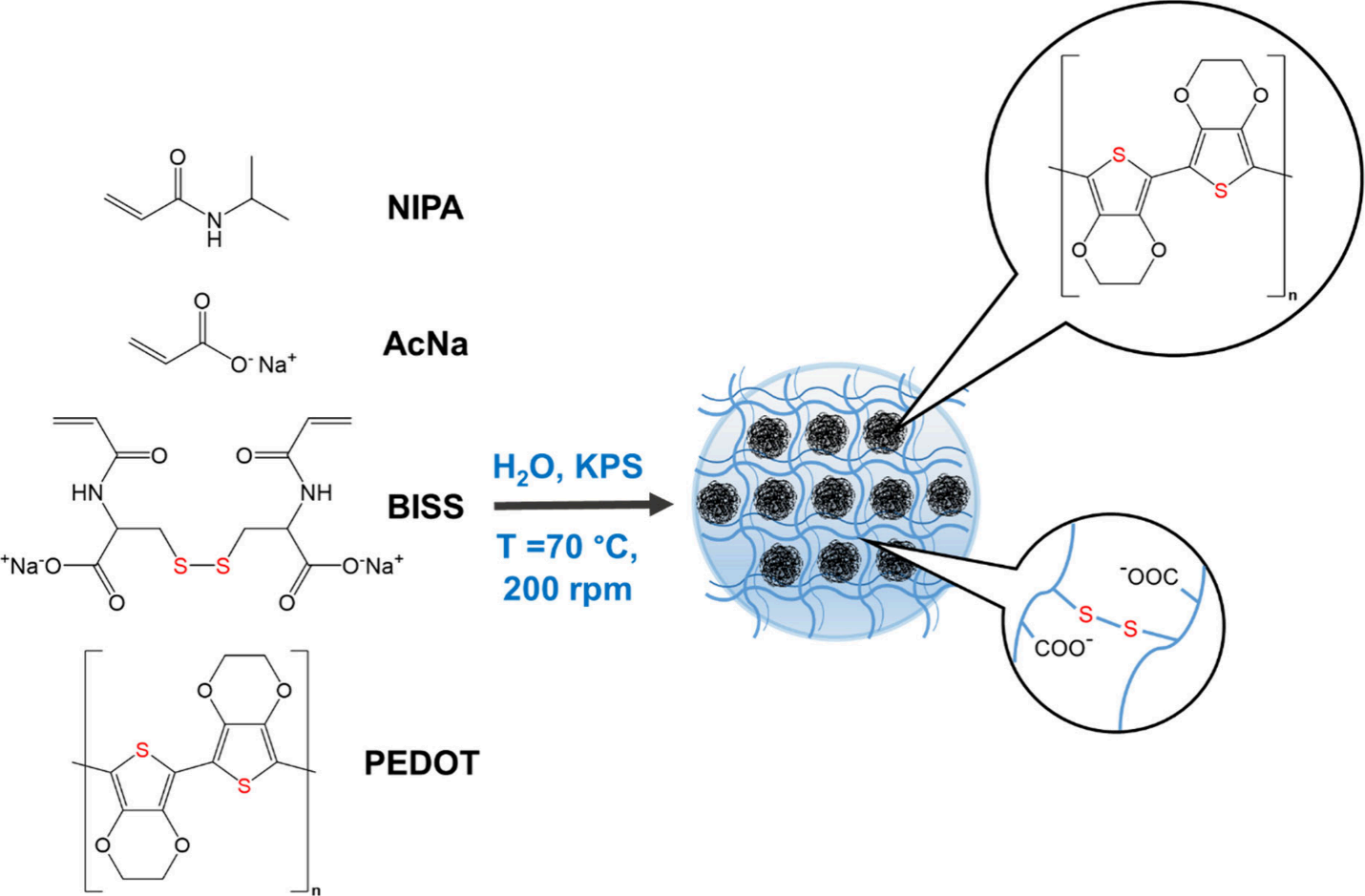
Scheme of p­(NIPA-BISS-AA)/PEDOT microgel synthesis.

### Instrumental

#### Dynamic Light Scattering (DLS)

The hydrodynamic diameter of microgel particles in aqueous dispersions was measured using dynamic light scattering with a Malvern Zetasizer Nano ZS (UK) instrument. A 4 mW helium–neon laser operating at a wavelength of 632.8 nm served as the light source, and measurements were taken at a backscattering angle of 173°. Prior to analysis, the samples were equilibrated at the desired temperature for 5 min.

#### Electrochemical Measurements

All electrochemical measurements were performed using a CH Instruments 400B potentiostat. A three-electrode system was employed, comprising a platinum wire as the counter electrode, a saturated silver/silver chloride electrode (Ag/AgCl/sat. KCl) as the reference electrode, and either a glassy carbon (GC) rod electrode or a gold quartz crystal microbalance with energy dissipation (QCM-D) electrode as the working electrode. All electrodes were placed in a glassy electrochemical cell or in modified manufacture QCM-D electrochemical cell. To minimize noise, the electrochemical cell was placed inside a Faraday cage.

#### QCM-D Measurements

QCM-D measurements were performed using a QEM 401 instrument from Q-Sense, Biolin Scientific. The device employed 4.95 MHz AT-cut gold-coated quartz crystals. Before experimentation, the electrode surface was cleaned by immersing it in a “hot-piranha” solution for 10 min to remove organic contaminants. This step was followed by rinsing with water and ethanol, and the electrode was then dried with a stream of argon gas. Finally, the electrode was placed in the modified QCM-D manufacturer electrochemical cell.

#### Scanning/Transmission Electron Microscopy (S/TEM)

To prepare the microgel samples for S/TEM analysis, a drop of the microgel particle aqueous suspension was deposited onto a Formvar-coated copper grid and left to air-dry. The samples were examined using a TALOS F200X microscope from Thermo Scientific. This instrument is equipped with advanced energy-dispersive X-ray spectroscopy (EDS) for signal detection and enables 3D chemical characterization through compositional mapping.

#### UV–Vis Measurements

Spectroscopic measurements were obtained with a UV–Vis spectrometer, model T-9100, Peak Instruments.

#### Nuclear Magnetic Resonance (NMR) Spectroscopy Measurements

The ^1^H NMR spectra for microgel characterization were recorded using a Bruker 300 spectrometer. Prior to analysis, the purified microgel solution was lyophilized and then dissolved in D_2_O.

## Results and Discussion

In the first step, the hydrodynamic diameter of the purified p­(NIPA-BISS-AA)/PEDOT microgel was examined using the Dynamic Light Scattering technique. The temperature-dependent plot of the hydrodynamic diameter is shown in Figure 3. As can be observed, at lower temperatures, the microgel is in a swollen state, with a measured diameter of approximately 200 nm at 20 °C. An increase in temperature above the volume phase transition temperature (approximately 33 °C) resulted in a decrease in the measured hydrodynamic diameter to about 65 nm at 45 °C. This behavior is characteristic of thermoresponsive p­(NIPA) microgels.

**Figure 3:**
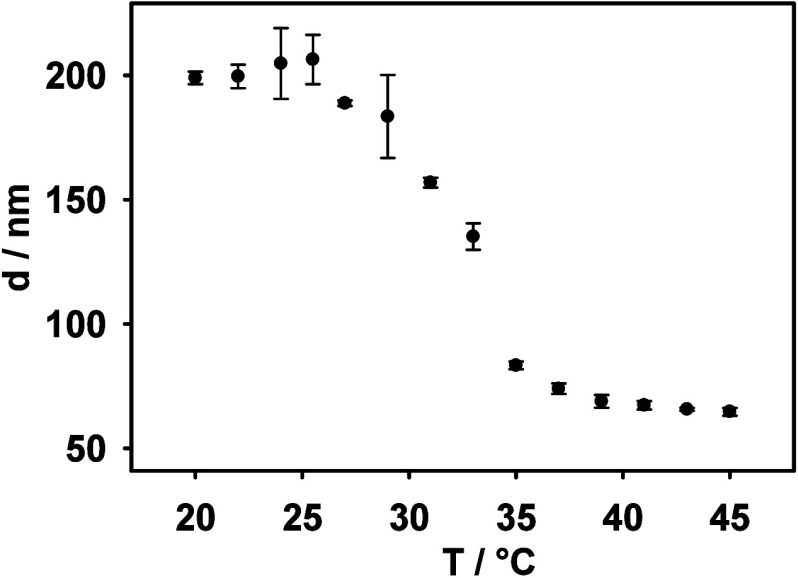
Temperature-dependent changes in the hydrodynamic diameter of p­(NIPA-BISS-AA)/PEDOT microgel.

The morphology of p­(NIPA-BISS-AA)/PEDOT microgel particles was analyzed using TEM, and the resulting micrograph is presented in Figure 4A. The particles exhibited a spherical shape with a smooth surface and an average diameter of approximately 67 nm. For comparison, the hydrodynamic diameter measured in the shrunken state using DLS was approximately 65 nm. A higher magnification image, highlighting individual microgel spheres, is shown in Figure 4B. Furthermore, compositional mapping of carbon and sulfur atoms was performed using the EDS technique. As depicted in Figure 4C, sulfur atoms were detected exclusively within the microgel particles, confirming the successful incorporation of PEDOT groups into the polymer network of the microgel.

**Figure 4:**
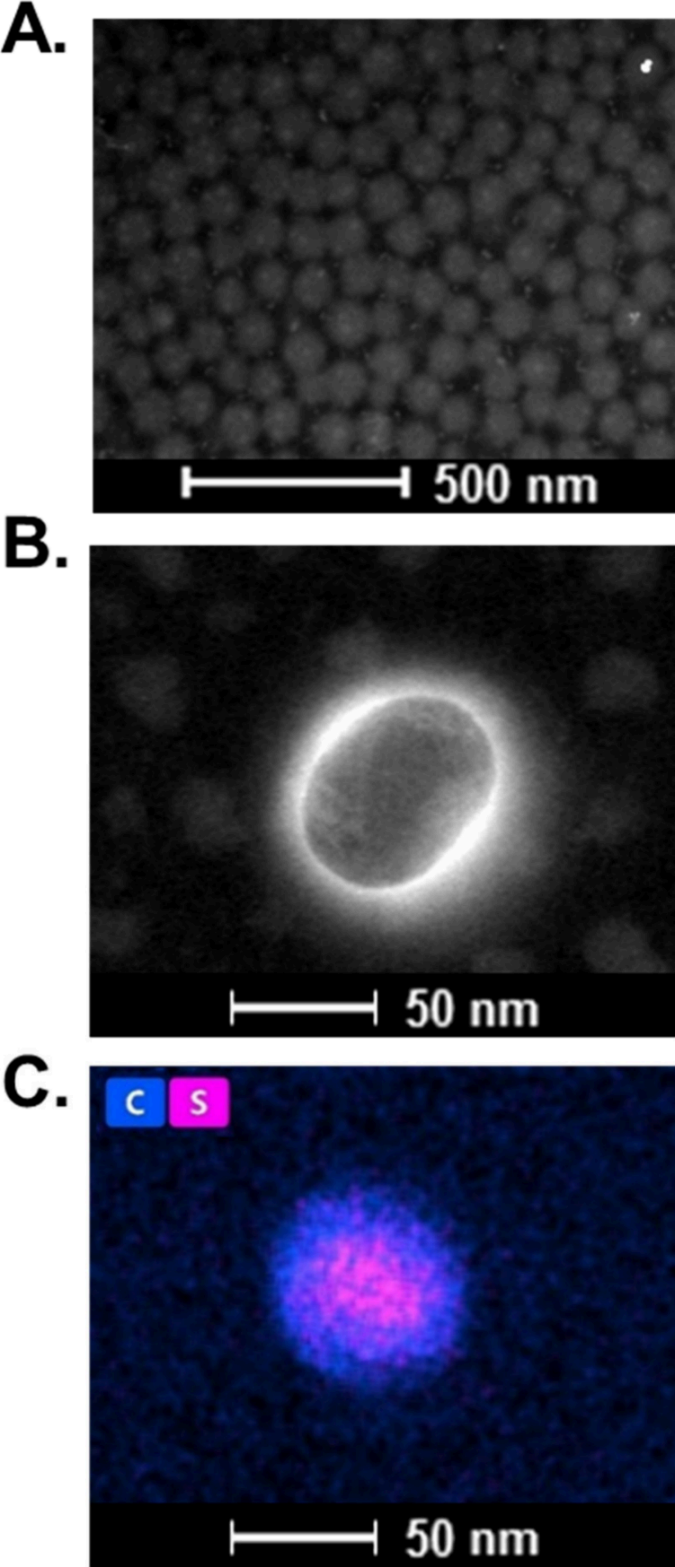
(A) TEM micrograph of p­(NIPA-BISS-AA)/PEDOT microgel spheres. (B) TEM micrograph of a single p­(NIPA-BISS-AA)/PEDOT microgel particle. (C) EDS compositional mapping of carbon and sulfur atoms in a single p­(NIPA-BISS-AA)/PEDOT microgel particle.

In the next step, the p­(NIPA-BISS-AA)/PEDOT microgel solution was electrochemically examined. For this purpose, 2 mL of the microgel solution, containing 0.02 M NaNO_3_ as the supporting electrolyte, was placed in a glassy electrochemical cell, and cyclic voltammograms were recorded. As shown in Figure 5, a characteristic pair of peaks for PEDOT moieties is present (red solid line), confirming that the PEDOT groups were successfully immobilized within the microgel polymer network and retained their electroactivity. In contrast, cyclic voltammograms recorded for the p­(NIPA-BISS-AA) microgel solution without PEDOT showed no electrochemical response (blue dashed line).

**Figure 5:**
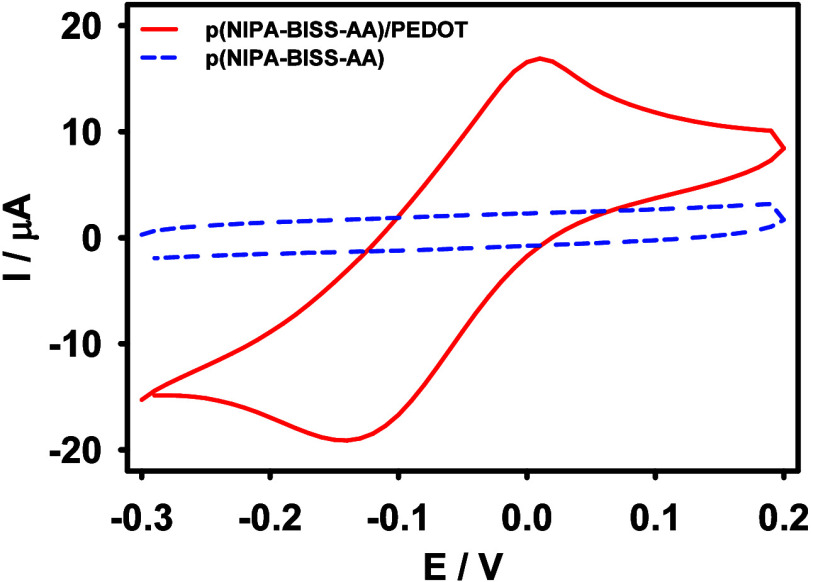
Cyclic voltammograms obtained in p­(NIPA-BISS-AA)/PEDOT (red solid line) and (NIPA-BISS-AA) (blue dashed line) microgel solutions. Supporting electrolyte: 0.02 M NaNO_3_. Working electrode: GC rod electrode, *T* = 25 °C, v = 50 mV/s.

The presence of disulfide bridges in the p­(NIPA-BISS-AA)/PEDOT microgel polymer network facilitated the attachment of microgel particles to the Au QCM-D electrode surface via chemisorption process. To achieve this, the Au QCM-D electrode was placed in the QCM-D electrochemical cell. Subsequently, 2 mL of Milli-Q water with an adjusted pH of 2 was added to the cell and heated to 40 °C. These conditions ensured that the microgel particles remained in a shrunken state, enabling the formation of a densely packed microgel monolayer on the electrode surface. Small portions of microgel solution (preheated to 40 °C) were then incrementally added to the QCM-D cell containing the electrode. The process was monitored in real time using the QCM-D technique. After each addition of 50 μL of microgel solution, frequency and energy dissipation shifts were allowed to stabilize before the next addition. The resulting frequency and energy dissipation shifts (measured for the third, fifth, and seventh overtones) are presented in Figure 6A. As shown, each addition of the microgel solution caused a decrease in the measured frequency and an increase in dissipation, corresponding to an increase in mass on the electrode surface. With successive additions, the changes in frequency and energy dissipation shifts became progressively smaller. After the seventh addition, no significant changes were observed, indicating that the electrode surface had reached saturation with a microgel monolayer. A schematic representation of the p­(NIPA-BISS-AA)/PEDOT microgel monolayer formation on the Au QCM-D electrode surface is provided in Figure 6B.

**Figure 6:**
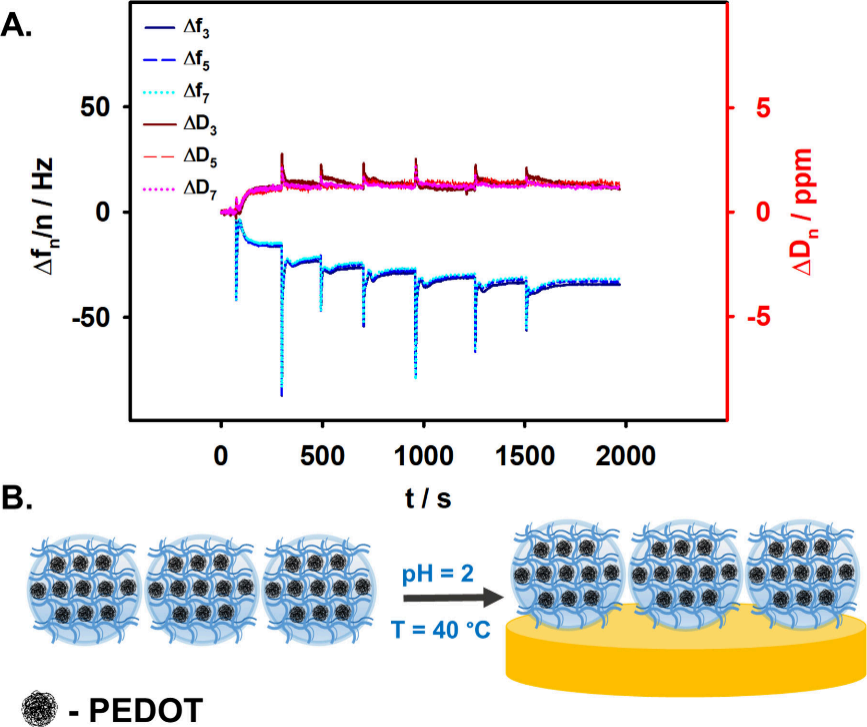
(A) Frequency and dissipation shifts (for the 3rd, 5th, and 7th overtones) recorded during the chemisorption of p­(NIPA-BISS-AA)/PEDOT microgel on the Au QCM-D electrode surface. (B) Schematic representation of the p­(NIPA-BISS-AA)/PEDOT microgel monolayer formation on the Au QCM-D electrode surface.

In the next step, the Au QCM-D electrode modified with the p­(NIPA-BISS-AA)/PEDOT microgel monolayer was subjected to electrochemical analysis. The modified electrode was placed in an electrochemical cell containing a supporting electrolyte (0.002 M NaNO_3_), and cyclic voltammograms were recorded. As shown in Figure 7, a characteristic pair of peaks associated with PEDOT moieties is present (red solid line). These peaks were absent for an electrode modified with the microgel monolayer without conductive polymer groups (blue dashed line). This confirms the successful attachment of the electroactive monolayer to the electrode surface.

**Figure 7:**
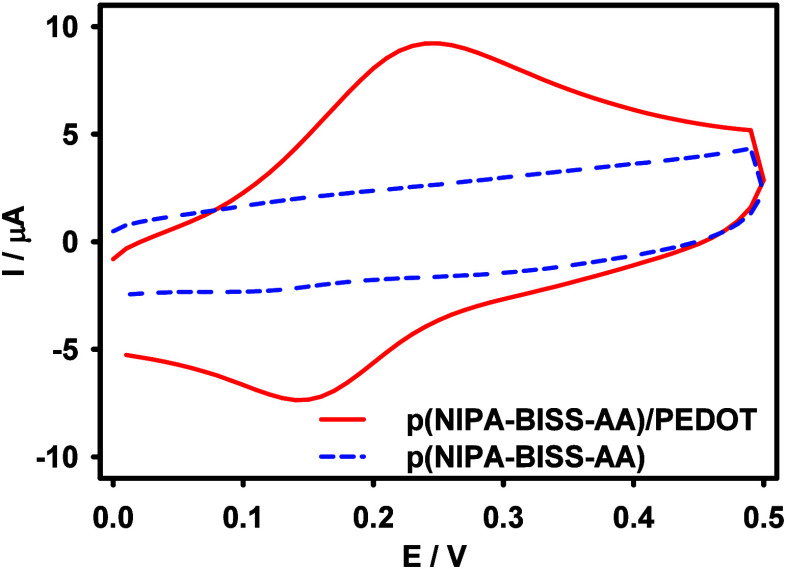
Cyclic voltammograms obtained with Au QCM-D electrode modified with p­(NIPA-BISS-AA)/PEDOT (red solid line) and p­(NIPA-BISS-AA) (blue dashed line) microgel monolayer electrode. Supporting electrolyte: 0.002 M NaNO_3_. *T* = 25 °C, v = 50 mV/s.

The introduction and immobilization of positively charged model drug molecules were achieved using the electrostatic interaction with negatively charged carboxylic groups in the p­(NIPA-BISS-AA)/PEDOT microgel monolayer on the Au QCM-D electrode surface. For this purpose, crystal violet (CV), a well-known positively charged dye often utilized as a model molecule, was selected. The process of introducing dye molecules into the p­(NIPA-BISS-AA)/PEDOT microgel monolayer was monitored using the QCM-D technique. In Figure 8A the typical frequency and dissipation shifts observed during this procedure was presented. Upon the addition of 50 μL of the dye solution (1 mM aqueous solution of crystal violet) to the QCM-D cell, prefilled with 1 mL of water, a notable decrease in frequency and a concurrent increase in dissipation were recorded. These changes indicate a mass increase on the electrode surface, confirming the successful immobilization of dye molecules within the microgel monolayer. Once frequency and dissipation values stabilized, subsequent additions of the dye solution were performed. However, these further additions resulted in negligible changes, indicating that the microgel monolayer had reached saturation with CV molecules. A plateau was achieved, suggesting no further binding capacity for the dye. Then, the modified with CV electrode covered with p­(NIPA-BISS-AA)/PEDOT monolayer was washed with water several times to remove unbound dye molecules. In Figure 8B a schematic illustration depicting the incorporation of dye molecules into the p­(NIPA-BISS-AA)/PEDOT microgel monolayer on the Au electrode surface.

**Figure 8:**
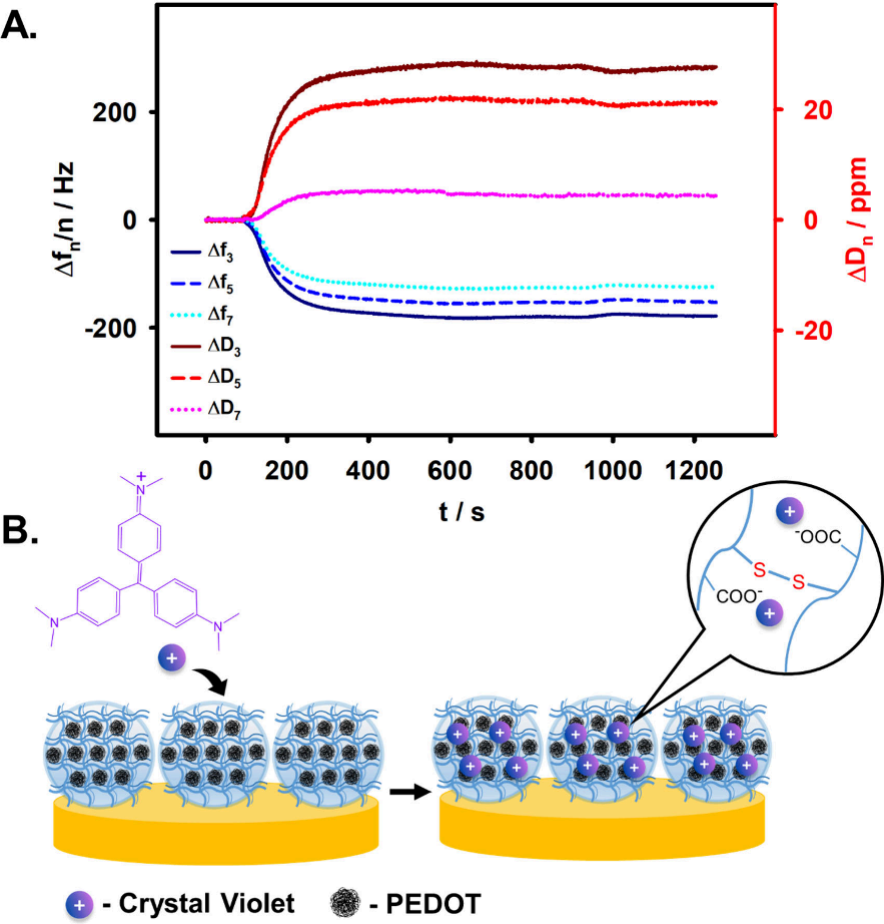
(A) Frequency and dissipation shifts (3rd, 5th, and 7th overtones) observed during the introduction of CV molecules into the p­(NIPA-BISS-AA)/PEDOT monolayer on the Au QCM-D electrode surface. (B) Schematic representation of CV incorporation into the p­(NIPA-BISS-AA)/PEDOT monolayer on the electrode surface.

In the next step, the electrochemically induced release of CV molecules was investigated. Oxidation of PEDOT within the microgel monolayer introduced positive charges into the polymer network, which was expected to weaken the electrostatic attraction between the dye and the microgel, while also creating repulsive interactions with the dye molecules. As a result, CV moieties were anticipated to be released into the surrounding environment. To examine this process, 3 mL of a 0.002 M NaNO_3_ supporting electrolyte solution was introduced into the QCM-D electrochemical cell containing the modified electrode. The release of the dye into the solution above the electrode was initiated using an electrochemical pulse. Prior to applying the potential, 1 mL of the solution was collected and transferred to a glass cuvette for UV–Vis spectroscopic measurement, after which the contents were returned to the cell. The release process was triggered by applying a potential of 0.3 V for 16 min using the chronoamperometric technique, with UV–Vis measurements taken every minute. As shown in Figure 9A (blue dots), the application of the potential led to an increase in the spectrophotometric signal, indicating the release of dye molecules from the microgel polymer network. After 5 min, the release curve reached a plateau, and no significant changes were observed thereafter, suggesting that most of the CV molecules had been released. For comparison, the release process was additionally observed in the absence of an applied potential. In this case, only a slight leakage of dye was observed during the first 3 min, with no significant changes recorded in the subsequent measurements (black squares). To determine the concentration of the released dye, a calibration curve was generated, illustrating the relationship between the spectrophotometric signal and crystal violet concentration, as it is presented as an inset in Figure 9A. The release profiles of CV from the p­(NIPA-BISS-AA)/PEDOT microgel monolayer on the electrode surface were calculated and are depicted in Figure 9B. The reversibility of dye molecule uptake following electrochemically induced release was also investigated. After CV release by applying a potential of 0.3 V for 5 min, the PEDOT moieties within the microgel monolayer were electrochemically reduced by applying 0 V for 5 min to the modified electrode. Subsequently, the dye uptake and release cycle was repeated three additional times. In the second release cycle, approximately 0.10 μM of CV was released, representing about 71% of the amount released during the first cycle (0.14 μM). In the third cycle, the release efficiency slightly increased to 75% of the initial value, and in the fourth cycle, it remained at 72% (inset in Figure 9C). These results indicate that after the initial release, the process stabilizes and is both reproducible and repeatable.

**Figure 9:**
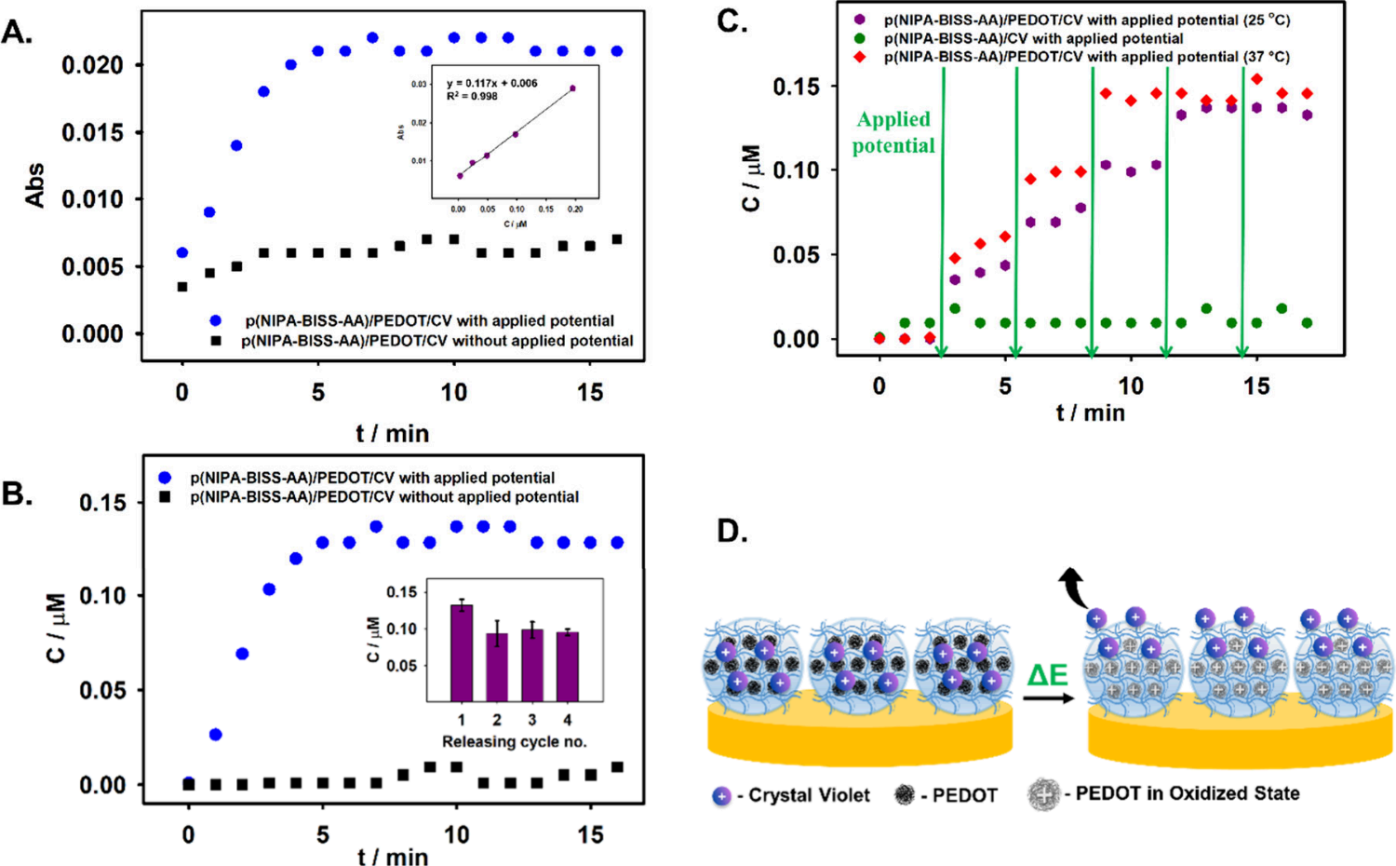
(A) CV release profiles from the p­(NIPA-BISS-AA)/PEDOT monolayer on the electrode surface under different conditions: continuously applied 0.3 V potential at 25 °C (blue dots) and without applied potential (black squares). Supporting electrolyte: 0.002 M NaNO_3_. Inset: Calibration curve for crystal violet dye. (B) CV concentration release profiles calculated based on the data presented in panel A. Inset: CV concentration released in each subsequent electrochemical release cycle. (C) CV concentration release profiles from the p­(NIPA-BISS-AA)/PEDOT monolayer on the electrode surface with pulsed potential application (0.3 V for 1 min) at 25 °C (purple hexes) and 37 °C (red diamonds). Additionally, the CV release profile from the p­(NIPA-BISS-AA) microgel monolayer with pulsed potential application (0.3 V for 1 min) is shown (green dots). Supporting electrolyte: 0.002 M NaNO_3_. (D) Schematic representation of the electrochemically induced release of CV molecules from the microgel monolayer on the electrode surface.

To demonstrate that the electrochemically induced release could be achieved under controlled conditions, measurements were performed using a pulsed applied potential and obtained results are shown in Figure 9C. This approach aimed to control the amount of crystal violet released. Initially, three spectrophotometric measurements of the solution above the electrode were taken without applying any potential; no spectrophotometric signal was observed, indicating that no dye was released. Subsequently, a potential of 0.3 V was applied to the electrode at 25 °C for 1 min, then stopped (indicated by purple hexes). During the 3 min pause after each potential application, three absorbance measurements were taken. This cycle of applying potential and measuring absorbance was repeated five times in total. The initial potential pulse resulted in an increase in measured absorbance, which was attributed to the release of a small amount of CV molecules from the polymer network. The following three pulses caused a gradual increase in absorbance, with the signal remaining stable after each pulse until the next one was applied. However, during the fifth cycle, no significant increase in absorbance was observed. This finding demonstrates that dye release can be precisely controlled by applying electrochemical pulses that induce partial oxidation of PEDOT. Furthermore, the total amount of dye released in this experiment is very similar to that obtained with continuous application of the potential.

Since the p­(NIPA-BISS-AA)/PEDOT microgel is thermosensitive, we investigated how temperature influences the release of CV from a microgel monolayer on the electrode surface. For this purpose, measurements were conducted at a temperature close to that of the human body (37 °C), when the microgel is in a shrunken state, as indicated by the data presented in Figure 3. It was observed that under these conditions (red diamonds), the release process was more efficient than at 25 °C. Specifically, the registered signal was higher at each step, and the plateau was reached after four electrochemical pulses, in contrast to five steps required at 25 °C. This can be explained by the fact that, in the shrunken state, the incorporated PEDOT is closer to the electrode surface, which facilitates more efficient electron transport during the oxidation process. Finally, we determined that the presence of PEDOT moieties in the microgel polymer network is essential for electrochemically induced release. To investigate this, an electrode coated with a p­(NIPA-BISS-AA) microgel monolayer containing immobilized CV molecules was subjected to pulsed potential application (0.3 V for 1 min). As shown in Figure 9C (green dots), the increase in the registered signal was minimal and independent of the applied potential, primarily attributed to the leakage of dye molecules from the microgel polymer network. This observation highlights the critical role of the electroactive PEDOT component in facilitating the release process. The total amount of dye released following continuous and pulsed potential application was approximately 0.14 μM at 25 °C and around 0.15 μM at 37 °C. A schematic representation of the electrochemically induced release of CV molecules from the p­(NIPA-BISS-AA)/PEDOT microgel monolayer on the Au electrode surface is provided in Figure 9D.

## Conclusion

A thermoresponsive hybrid microgel was successfully synthesized via precipitation polymerization. The polymer network of the microgel, based on *N*-isopropylacrylamide and acrylic acid cross-linked with *N,N*′-bisacryloylcystine, effectively immobilized PEDOT, imparting electroactive properties. The thermally induced size transitions of the microgel were investigated using dynamic light scattering (DLS), revealing a significant temperature responsiveness: in its swollen state, the microgel reached a size of approximately 200 nm, while heating caused it to shrink dramatically to around 60 nm. The morphology and elemental composition of the microspheres were characterized using transmission electron microscopy (TEM) and energy-dispersive X-ray spectroscopy (EDS). Electrochemical studies conducted via cyclic voltammetry confirmed the presence of characteristic signals corresponding to PEDOT groups, highlighting the electroactive properties of the microgel. A unique feature of the microgel was the incorporation of disulfide bridges within its polymer network, enabling chemisorption onto gold electrode surfaces. This surface modification process was monitored in real-time using quartz crystal microbalance with dissipation monitoring (QCM-D). Furthermore, the carboxylic groups in the microgel’s polymer matrix facilitated the adsorption and accumulation of positively charged molecules, exemplified by the model dye crystal violet. This process was also tracked with QCM-D. The potential for controlled dye release via electrochemical stimulation was thoroughly investigated. By applying an electrochemical trigger, the release of crystal violet from the polymer network was successfully achieved and monitored using UV–Vis spectroscopy. The mechanism of release was linked to the electrochemical oxidation of PEDOT groups, which generated positive charges within the microgel structure. This disrupted the electrostatic interactions between the dye molecules and the polymer chains, facilitating the controlled release of the dye into the surrounding environment. Notably, the process occurred exclusively when an appropriate oxidation potential was applied, and the quantity of the released substance could be finely tuned by adjusting the electrochemical signal. As a result, the p­(NIPA-BISS-AA)/PEDOT microgel monolayer demonstrates exceptional potential as a drug carrier platform for advanced, electrochemically controlled release systems. A specially possibility to precisely controlled amount of release substance with electrochemical signal improves this possibility. This innovative material holds significant promise for applications in biomedicine, e.g. the development of next-generation electrochemical devices, such as intrabody delivery implants or transdermal delivery platforms.
